# Textural Properties of Carbopol^®^ Gel with Curcumin and Curcumin–HPβCD Inclusion Complex and Biological Activities

**DOI:** 10.3390/gels12010077

**Published:** 2026-01-16

**Authors:** Maja Urošević, Vesna Nikolić, Vesna Savić, Tatjana Mihajilov-Krstev, Ivana Gajić, Ana Dinić, Milica Martinović, Ljubiša Nikolić

**Affiliations:** 1 Faculty of Technology, University of Niš, Bulevar Oslobodjenja 124, 16000 Leskovac, Serbia; maja@tf.ni.ac.rs (M.U.); nikolicvesna@tf.ni.ac.rs (V.N.); ivana@tf.ni.ac.rs (I.G.); anatacic@tf.ni.ac.rs (A.D.); 2 Department of Pharmacy, Faculty of Medicine, University of Niš, Bulevar Dr Zorana Djindjica 81, 18000 Niš, Serbia; vesna.savic@medfak.ni.ac.rs (V.S.); milica.martinovic@medfak.ni.ac.rs (M.M.); 3 Department of Biology and Ecology, Faculty of Sciences and Mathematics, University of Niš, Višegradska 33, 18000 Niš, Serbia; tatjana.mihajilov-krstev@pmf.edu.rs

**Keywords:** Carbopol^®^ gel, curcumin, curcumin:2-hydroxypropyl-β-cyclodextrin inclusion complex, texture analysis, antimicrobial activity, antioxidant activity

## Abstract

The aim of this study was to prepare curcumin/2-hydroxypropyl-β-cyclodextrin (HPβCD) inclusion complex, evaluate the antioxidant and antimicrobial activities of curcumin and curcumin in inclusion complex, as well as to examine the effect of curcumin and curcumin inclusion complex on the textural properties of Carbopol^®^ gel mixture. Curcumin/2-hydroxypropyl-β-cyclodextrin inclusion complex was prepared using the co-precipitation method in a molar ratio of 1:1. The antioxidant activity of curcumin and curcumin in inclusion complex was determined using DPPH, ABTS, and FRAP methods. The micro-dilution method was used to examine in vitro the antimicrobial activity of curcumin and curcumin in inclusion complex. The textural, rheological, and morphological properties of the samples (Gel 1- Carbopol^®^ gel; Gel 2- Carbopol^®^ gel with dispersed curcumin inclusion complex; Gel 3- Carbopol^®^ gel with dispersed curcumin) were examined. The textural properties were evaluated using a texturometer CT3 Texture Analyzer by means of a Texture Profile Analysis (TPA) test. The results showed that curcumin in inclusion complex had higher antioxidant and antimicrobial activity. The SEM confirms the presence of curcumin and inclusion complex in Carbopol^®^ gel. The rheological analysis confirmed that the structural integrity of Carbopol^®^ gel was preserved. The highest swelling degree is achieved by Gel 3 formulation. The in vitro release of curcumin from Gel 2 and Gel 3 occurs at the rates of 0.414 µg/h·g_gel_ and 0.376 µg/h·g_gel_, respectively. The textural analysis showed that adding curcumin or its inclusion complex did not significantly change the properties of Carbopol^®^ gel, indicating potential for topical use.

## 1. Introduction

Carbopol^®^ is an acrylic polymer widely used for making gels. It is non-toxic and non-irritating with repeated use, which makes it suitable for developing gel formulations [1]. Carbopol^®^ is the main component in drug delivery systems for buccal, ocular, and dermatological topical applications. Bioadhesive and thermostable Carbopol^®^ hydrogels are characterized by high viscosity and compatibility with biologically active substances [2,3,4,5]. Biologically active substances of natural origin are a subject of great interest in many areas of current pharmaceutical practice. Therefore, many scientific teams have investigated the possibilities of incorporating non-synthetic substances into topical application systems [6]. Various formulations have been designed using natural substances [7,8,9] such as curcumin or diferuloylmethane, a natural polyphenol obtained from the rhizome of *Curcuma longa*. They have antioxidant, antimicrobial, and anti-inflammatory effects, and are used as wound healing agents [10,11,12]. The topical application of curcumin is useful to ensure a localized effect in skin diseases [13,14,15]. Although herbal medicines are popular due to their safety and mild properties, physicochemical properties such as solubility and permeability can have significant impact on their efficacy. Better solubility and an increase in effectiveness of active ingredients is achieved by developing new strategies for creating formulations with modern carriers [16]. Formulations that include liposomes, micelles, cyclodextrin complexes, and hydrogels as carriers can significantly improve local availability of a poorly water-soluble drug [17]. Cyclodextrins can form molecular inclusion complexes with lipophilic compounds, improving water solubility, dispersibility, and absorption of active components. The bioavailability of a curcumin formulation with γ-cyclodextrin [18], hydroxypropyl-β-cyclodextrin [19,20], and β-cyclodextrin [21] has been investigated and numerous clinical studies on the topical application of curcumin have been delivered [22,23,24]. The application of numerous curcumin-based topical formulations developed for wound therapy remains limited due to its poor solubility in aqueous media, chemical instability and insufficient local bioavailability, which significantly reduces its therapeutic efficacy. A study by Shefa et al. describes the development of a biocompatible hydrogel based on polyvinyl alcohol (PVA) and TEMPO-oxidized cellulose nanofibers (TOCN) by incorporating curcumin (Cur) to enhance wound healing. The hydrogel, which was physically crosslinked using a freeze–thaw method, exhibited a porous structure, sustained curcumin release and demonstrated good biocompatibility in vitro. In a full-thickness skin wound model in rats, the hydrogel accelerated wound closure and promoted the formation of new epithelium, granulation tissue and collagen deposition, when compared to control groups. These results suggest that TOCN-PVA-Cur hydrogel could serve as an effective local curcumin delivery system for wound therapy [25]. The synthesis and characterization of alginate gel beads with embedded zeolite structures for encapsulating hydrophobic curcumin, aimed at improving its dispersibility and enabling controlled release of the active compound, were described by Ciarleglio et al. The results indicated that these pH-responsive composite carriers are promising systems for the oral delivery of lipophilic compounds such as curcumin [26]. Various types of nanocarriers, including lipid-based nanomaterials, polymeric nanocarriers, inorganic nanomaterials, and hybrid systems, have been developed to enhance the delivery of curcumin to tumor tissue and are extensively reviewed by Yan et al. [27]. Sarfaraz et al. developed a novel system for enhancing wound healing that combines curcumin (Cur)—a naturally bioactive compound with anti-inflammatory and antioxidant properties, silver (Ag)—known for its strong antimicrobial activity and metal–organic frameworks (MOFs) as porous nanostructures facilitating drug loading and controlled release in the wound. The study demonstrated that the conjugated Cur-Ag-MOF nanocomposites significantly improve wound healing through the synergistic action of curcumin and silver, prolonged bioactive substance release, inflammation reduction and tissue regeneration stimulation [28]. Deng et al. provided a comprehensive review of topical systems designed for curcumin-based wound therapy, including hydrogel dressings, nanofibers, films, lipidic and micellar systems, and nanoemulsion gels. The aim of these formulations is to maintain therapeutic curcumin levels at the wound site to exploit its anti-inflammatory, antioxidant, and antimicrobial properties, as well as its capacity to stimulate cell proliferation and angiogenesis. Hydrogels are among the most frequently used topical systems due to their ability to maintain a moist wound environment and enable sustained curcumin release [29]. Specifically, chitosan and oxidized alginate-based hydrogels [30], curcumin-loaded micellar hydrogels [31], dual-loaded hydrogels with chitosan and gelatin [32], thermosensitive polymeric hydrogels [33], and hydrogels incorporating liposomal carriers to enhance skin penetration [34] have been described. Song et al. developed a Carbopol^®^ 940-based hydrogel containing a curcumin-loaded micellar system (MPEG–PVL–PCL) for topical application in inflammation treatment and wound healing. Their results demonstrated the potential of this system for treating full-thickness skin wounds due to the combination of local efficacy and antioxidant and anti-inflammatory properties of curcumin, and the advantages of Carbopol^®^ hydrogel as a carrier [35]. Senthil et al. developed a dual-loaded gel for oral wounds, including post-dental procedure wounds. The formulation contains curcumin (anti-inflammatory and pro-regenerative agent) and ciprofloxacin (antibiotic) incorporated into a Carbopol^®^ gel base. Preliminary studies demonstrated encouraging antimicrobial, anti-inflammatory, regenerative, and clinical effects [36]. The mechanical properties (hardness, cohesiveness, adhesiveness, resilience, elasticity) of semi-solid topical formulations are significant for their applicability at the administration site, drug delivery, and acceptance by consumers, which overall has impact on the effectiveness of the formulation. A texture analysis is a technique that provides an objective insight into the mechanical properties of pharmaceutical and cosmetic products and has been intensively used in recent years [37,38]. Although the described systems provide advanced functionalities, they often require complex synthesis or formulation procedures. In contrast, Carbopol^®^ 940—curcumin/2-hydroxypropyl-β-cyclodextrin system represents a simple, practical, and more accessible alternative for topical application. Based on the available literature, no formulation has yet been reported that combines Carbopol 940^®^ gel—a biocompatible, non-toxic, and widely used polymer in dermatological preparations—with curcumin/2-hydroxypropyl-β-cyclodextrin inclusion complex for topical use. 2-hydroxypropyl-β-cyclodextrin is also safe for dermal application and is frequently employed in cosmetic and pharmaceutical systems due to its ability to enhance the solubility of lipophilic molecules. The synergy of these two excipients makes the formulation particularly promising: the inclusion complex ensures improved solubility, stability, and bioavailability of curcumin, while Carbopol 940^®^ provides a stable gel matrix, controlled release, good adhesion, and maintenance of a moist environment necessary for optimal wound healing. Such a system is especially suitable for the treatment of superficial and chronic wounds, as it enables localized, sustained, and targeted delivery of curcumin with minimal risk of skin irritation.

The aim of this study was to prepare curcumin/2-hydroxypropyl-β-cyclodextrin inclusion complex in order to increase the solubility of curcumin and its bioavailability, to evaluate the antioxidant and antimicrobial activity of curcumin and curcumin in inclusion complex, as well as the effect of curcumin and curcumin inclusion complex on the textural properties of Carbopol^®^ gel mixture.

## 2. Results and Discussion

**Figure 1:**
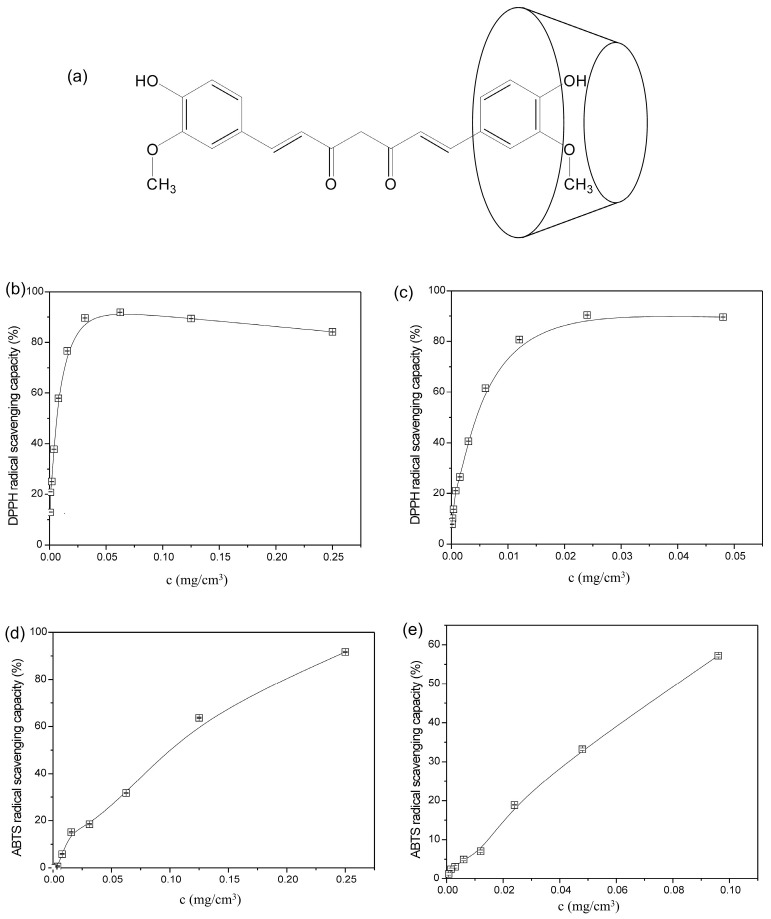
Curcumin incorporation into 2-hydroxypropyl-β-cyclodextrin and antioxidant activity: (**a**) schematic representation of curcumin inclusion into 2-hydroxypropyl-β-cyclodextrin, (**b**) DPPH radical scavenging for free curcumin, (**c**) DPPH radical scavenging for curcumin in inclusion complex, (**d**) ABTS radical scavenging for free curcumin, (**e**) ABTS radical scavenging for curcumin in inclusion complex.

Curcumin/2-hydroxypropyl-β-cyclodextrin inclusion complex was successfully prepared using the co-precipitation method according to the procedure followed in a study by Nikolić et al. [39]. Nikolić et al. proved the incorporation of curcumin in the cavity of 2-hydroxypropyl-β-cyclodextrin using FTIR, ^1^H-NMR, XRD, DSC, and SEM methods. The phase solubility test showed that by incorporating curcumin into 2-hydroxypropyl-β-cyclodextrin, the solubility of curcumin in water increased 1237.18 times and that the molar ratio of curcumin and 2-hydroxypropyl-β-cyclodextrin in the inclusion complex was 1:1 [39]. Figure 1 shows a potential way of incorporating curcumin into the cavity of cyclodextrin since, according to Tang et al., the width of curcumin molecule is 0.6 nm and the length is 1.9 nm, thus allowing the aromatic part of curcumin to enter the 0.74 nm wide cavity of cyclodextrin [40].

### 2.1. Antioxidant Activity of Curcumin and Curcumin/2-Hydroxypropyl-β-Cyclodextrin Inclusion Complex

#### 2.1.1. DPPH Test

The antioxidant activity of curcumin and curcumin/2-hydroxypropyl-β-cyclodextrin inclusion complex was evaluated using a DPPH test and the results obtained after a 20 min—incubation are shown in Figure 1b,c.

The DPPH radical scavenging capacity of curcumin and curcumin in curcumin/2-hydroxypropyl-β-cyclodextrin inclusion complex increases with an increase in the concentration of the used solution (Figure 1b,c). When using a curcumin solution at a concentration of 0.03125 mg/cm^3^, the capacity of neutralized DPPH radicals is 89.53%, while an increase in the concentration of curcumin solution to 0.0625 mg/cm^3^ neutralizes 91.84% of DPPH radicals, which also represents the maximum value of the DPPH radical scavenging capacity. The EC_50_ value, which represents the concentration of curcumin that neutralizes 50% of the initial concentration of DPPH radicals, is 0.00677 ± 3.33 × 10^−4^ mg/cm^3^. The percentage of neutralized DPPH radicals in curcumin/2-hydroxypropyl-β-cyclodextrin inclusion complex solution increases sharply after 20 min of incubation with an increase in curcumin concentration. The solution of curcumin/2-hydroxypropyl-β-cyclodextrin inclusion complex, where the concentration of curcumin is 0.012 mg/cm^3^, neutralizes 80.65% of DPPH radicals. The maximum value of DPPH radical scavenging capacity is 90.31% and is achieved at a curcumin concentration of 0.024 mg/cm^3^ in curcumin/2-hydroxypropyl-β-cyclodextrin inclusion complex solution. The EC_50_ value for curcumin in curcumin/2-hydroxypropyl-β-cyclodextrin inclusion complex solution is 0.0044 ± 8.22 × 10^−5^ mg/cm^3^. The synthetic BHT antioxidant exhibits an EC_50_ value of 0.021 mg/cm^3^ after a 20 min incubation with the DPPH radical [41]. The EC_50_ values for BHT, curcumin, and curcumin/2-hydroxypropyl-β-cyclodextrin inclusion complex decrease in the order as follows 0.021 mg/cm^3^ > 0.00677 mg/cm^3^ > 0.0044 mg/cm^3^, respectively, and show that the antioxidant activity of curcumin increased about as many as 1.52 times after complexation with 2-hydroxypropyl-β-cyclodextrin.

#### 2.1.2. ABTS Test

The dependence of ABTS radical scavenging capacity on the concentration of pure curcumin and curcumin in curcumin/2-hydroxypropyl-β-cyclodextrin inclusion complex after a 6 min incubation is shown in Figure 1d,e.

The capacity of ABTS radical neutralization of curcumin and curcumin in curcumin/2-hydroxypropyl-β-cyclodextrin inclusion complex increases with higher concentration of the solution after a 6 min of incubation (Figure 1d,e). The maximum value of ABTS radical scavenging capacity of pure curcumin is 91.69% and is achieved at a curcumin concentration of 0.25 mg/cm^3^, while the maximum value of scavenging capacity of 56.92% is achieved at a curcumin concentration of 0.096 mg/cm^3^ in curcumin/2-hydroxypropyl-β-cyclodextrin inclusion complex solution. In the study by Bayomi et al., where an ABTS test was used, the maximum value of ABTS radical scavenging capacity of curcumin is 87.64% and is slightly lower than the value achieved in this research [42]. The EC_50_ value, which represents the concentration of curcumin that neutralizes 50% of the initial ABTS radical concentration, is 0.098 ± 3 × 10^−4^ mg/cm^3^, while for curcumin in curcumin/2-hydroxypropyl-β-cyclodextrin inclusion complex solution it is 0.083 ± 1 × 10^−3^ mg/cm^3^. The results of the ABTS test indicate that the antioxidant activity of curcumin increases through complexation with 2-hydroxypropyl-β-cyclodextrin.

#### 2.1.3. FRAP Test

A FRAP test is a colorimetric test based on determining the capacity of antioxidants to donate electrons in the process of reducing ferric-tripyridyl triazine to ferrous-tripyridyl triazine at a low pH [43]. The ferrous-tripyridyl triazine complex solution is blue in color and has an absorbance maximum at 593 nm. The results of the FRAP test show that curcumin in curcumin/2-hydroxypropyl-β-cyclodextrin inclusion complex at a concentration of 0.024 mg/cm^3^ demonstrates a higher reducing ability (FRAP value 532.53 mg Fe^2+^/g_curcumin_) compared to pure curcumin at a concentration of 0.125 mg/cm^3^ (FRAP value 520.63 mg Fe^2+^/g_curcumin_).

The results obtained using DPPH, ABTS and FRAP tests indicate that the antioxidant activity of curcumin has increased along with the complexation with 2-hydroxypropyl-β-cyclodextrin. The reason for this is probably the increased solubility of curcumin, which was achieved through complexation.

### 2.2. Antimicrobial Activity of Curcumin and Curcumin/2-Hydroxypropyl-β-Cyclodextrin Inclusion Complex

**Figure 2:**
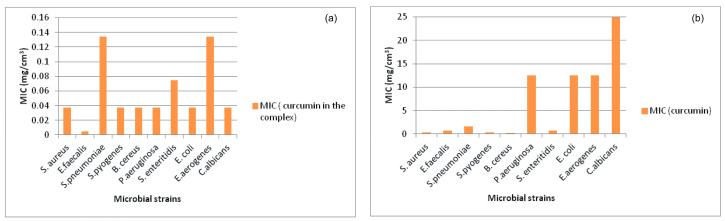
Graphical depiction of antimicrobial activity results: (**a**) antimicrobial activity (MIC) of curcumin in the complex, (**b**) antimicrobial activity (MIC) of curcumin.

The results obtained by evaluating the antimicrobial activity of pure curcumin in the concentration range of 0.003–25.0 mg/cm^3^, the prepared curcumin/2-hydroxypropyl-β-cyclodextrin inclusion complex, in which the concentration of curcumin is in the range from 5.8 × 10^−4^ to 4.82 mg/cm^3^, and the standard antimicrobial agents are shown in Table 1. A graphical representation of the antimicrobial results is given in Figure 2.

Based on the findings shown in Table 1, it can be concluded that pure curcumin and curcumin in curcumin/2-hydroxypropyl-β-cyclodextrin inclusion complex exhibit antibacterial activity against all tested Gram (+) and Gram (–) bacterial strains and antifungal activity against the tested *C. albicans* strain. Pure curcumin has an inhibitory effect on all tested strains in the MIC range (0.19–12.5 mg/cm^3^) and is bactericidal at an MBC value of 25 mg/cm^3^ on *S. aureus*, *E. faecalis* and *S. pyogenes* strains, while for other strains the MBC value is higher than 25 mg/cm^3^. The obtained results show that the antibacterial effect of curcumin is more significant on Gram (+) bacteria, which is in accordance with the reference data [44]. *B. cereus* is the most sensitive to the action of pure curcumin and the MIC value is 0.19 mg/cm^3^. *B. cereus* is an optionally anaerobic Gram (+) toxin-producing bacterium found in soil, flora, and food. It usually causes intestinal ailments with nausea, vomiting, and diarrhea. In the study by Wang et al., the findings of the research showed that curcumin has the potential to be used as a natural preservative to control the growth of contaminant microorganisms (*B. cereus*) and extend the shelf life of fresh millet noodles. The MIC value of curcumin against *B. cereus* is 0.125 mg/cm^3^ [45] and is approximate to the value obtained in this study. The results shown in Table 1 indicate an increase in the antimicrobial (antibacterial and antifungal) activity of curcumin through complexation with 2-hydroxypropyl-β-cyclodextrin. The MIC values for curcumin in curcumin/2-hydroxypropyl-β-cyclodextrin inclusion complex are in the range of 0.005–0.134 mg/cm^3^, while the MBC value is 4.82 mg/cm^3^ for the strains of *S. aureus*, *E. faecalis*, and *S. pyogenes*, whereas for other strains the MBC value is higher than 4.82 mg/cm^3^ (Table 1). The lowest MIC value (5 μg/cm^3^) for curcumin in curcumin/2-hydroxypropyl-β-cyclodextrin inclusion complex was achieved for the *E. faecalis* strain and is very close to the MIC value (3.91 μg/cm^3^) for the tested antibiotic Chloramphenicol and even 156 times lower than the MIC value obtained (0.78 mg/cm^3^) for pure curcumin for the same microbial strain. The lowest MIC value (5 μg/cm^3^) for curcumin in curcumin/2-hydroxypropyl-β-cyclodextrin inclusion complex is 38 times lower than the lowest value for pure curcumin (0.19 mg/cm^3^), while the lowest MBC value (4.82 mg/cm^3^) for curcumin in curcumin/2-hydroxypropyl-β-cyclodextrin inclusion complex is five times lower than the lowest MBC value (25 mg/cm^3^) for pure curcumin. The higher microbiological effectiveness of curcumin in inclusion complex is probably the result of an increase in its solubility in water.

### 2.3. Texture Analysis

**Figure 3:**
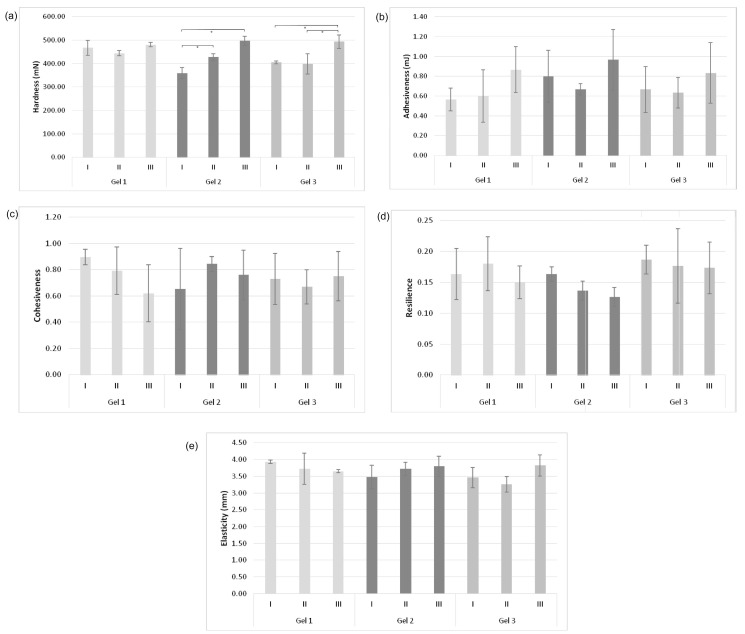
Texture analysis of Gel 1, Gel 2, and Gel 3: (**a**) hardness, (**b**) adhesiveness, (**c**) cohesiveness, (**d**) resilience, and (**e**) elasticity measured immediately after preparation (I), 7 days (II), and 14 days (III) after preparation. Statistically significant differences are indicated by * (*p* < 0.05).

**Figure 4:**
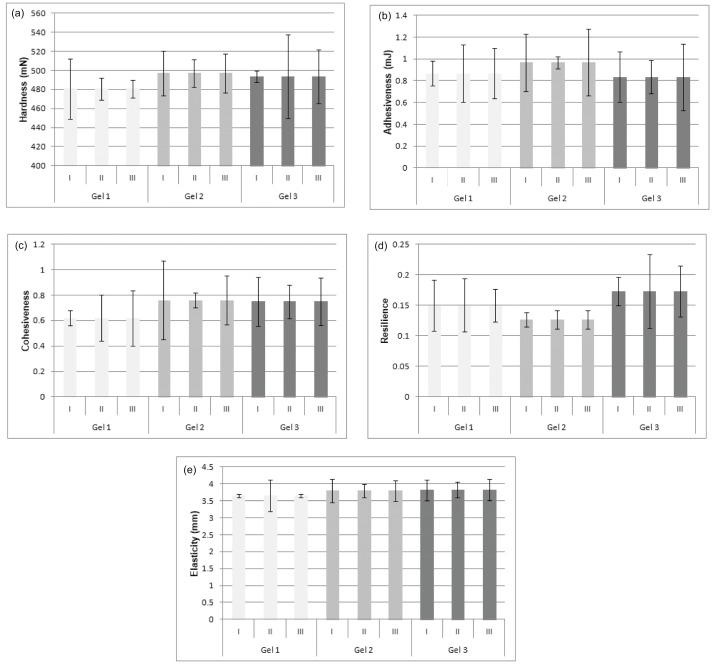
Texture analysis of Gel 1, Gel 2, and Gel 3: (**a**) hardness, (**b**) adhesiveness, (**c**) cohesiveness, (**d**) resilience, and (**e**) elasticity measured over 21 days (I), 28 days (II), and 35 days (III) after preparation.

Topical drug delivery is becoming increasingly popular due to its convenience and affordability [46]. Topical formulations are available in various forms such as ointments, creams, gels, and lotions [47]. Advantages of using a gel formulation compared to other semi-solid dosage forms are its non-greasy nature, easy application, and skin removal, as well as its excellent spreadability [15]. Gel formulations consist of active substances, cross-linking agents, and other additives. The concentration of the crosslinking agent must be appropriate since it is one of the parameters that affects the nature and physical stability of gel, which in turn can affect the absorption of active substances on the skin [48]. In the study by Shuja et al., a topical gel was prepared with curcumin-β-cyclodextrin inclusion complex in combination with aloe vera. Carbopol^®^, carboxy methyl cellulose and guar gum were used in different concentrations as crosslinking agents. The prepared formulations were tested against various parameters such as physical appearance, spreadability, drug content, pH, viscosity, and in vitro permeation. It was concluded that the permeability of curcumin improved after its complexation with β-cyclodextrin. An aloe vera based topical gel with inclusion complex of curcumin, 1% Carbopol 940^®^, and 10% propylene glycol showed maximum effectiveness [15]. Patel et al. showed that a topical curcumin gel prepared with Carbopol^®^ showed a better percentage of inflammation inhibition compared to a gel prepared with hydroxypropyl cellulose [13]. A gel prepared with Carbopol 934^®^ and propylene glycol containing 1% curcumin can be used to promote wound healing [49]. When developing topical dosage forms, it is important to define several important parameters that contribute to the ultimate acceptability of the product by patients and the clinical efficacy of the product. This includes optimal mechanical properties (e.g., ease of removal of the product from the container, good lubricity on the skin or mucosa), good bioadhesion, acceptable viscosity, drug release, and absorption. Therefore, it is important to examine effects on the rheology of products, and, hence, on their clinical performance [50]. Textural properties are an important parameter for the optimization of topical formulations. These properties will affect the applicability of the formulation at the administration site and therapy outcome [37]. The operating principle of the texture analyzer used for these purposes is based on immersing the probe in the sample while simultaneous measuring the forces with which the probe acts on the formulation, the distance the probe travels during immersion in the formulation and the time required for movement. On the basis of measured parameters, a force-distance graph is generated, from which the data can be further interpreted and the values of the mechanical parameters obtained [51]. The texture parameters (hardness, adhesiveness, cohesiveness, resilience, and elasticity) of pure Carbopol^®^ gel (Gel 1), gel with curcumin inclusion complex (Gel 2), and curcumin (Gel 3) were measured immediately after preparation (day 0), 14, 21, 28, and 35 days to assess their stability over time (Figure 3 and Figure 4).

Hardness is a parameter that represents the resistance of the tested sample to the applied pressure force [3,52]. The hardness of the samples was measured during the first immersion of the probe and the results are shown in Figure 3a.

By analyzing the obtained values (Figure 3a), a statistically significant increase in the hardness of the gel sample with curcumin inclusion complex (Gel 2) and the gel with curcumin (Gel 3) was observed after 14 days from the time of preparation. The curcumin inclusion complex and curcumin affected structuring of the gel, while the hardness values did not change significantly in statistical terms in pure Carbopol^®^ gel (Gel 1). At the time of preparation, the presence of curcumin inclusion complex and curcumin in the gel resulted in a decrease in the hardness of the gel (Gel 2 and Gel 3) compared to the hardness of pure Carbopol^®^ gel (Gel 1), so that after 14 days from the time of preparation, all gels (Gel 1, Gel 2, and Gel 3) had almost the same hardness values, which indicates the positive influence of the bioactive substance on the stability of hydrogel. Adhesiveness represents the force needed to overcome the attracting forces that arise between the probe and the formulation, that is, it describes the adhesiveness of the tested formulations [3,52]. Figure 3b shows the adhesiveness values of the tested gel samples (Gel 1, Gel 2, and Gel 3) over a period of 14 days.

The test results (Figure 3b) showed that there were no statistically significant differences in the adhesiveness values of all three tested gel samples (Gel 1, Gel 2, and Gel 3), which indicates that the presence of curcumin inclusion complex and curcumin in the gel did not affect the adhesiveness of the gel. Cohesiveness is a parameter that determines whether the structural integrity of the formulation will change due to the action of force on the formulation. It is desirable for the adequate applicability of the formulation, that the structural integrity of the formulation not be damaged due to the action of the force. Resilience is a parameter that represents the resistance of the formulation, that is, it determines how the formulation will cope with the stress which it is exposed to. Resilience indicates the tendency of the formulation to return to its initial position after deformation. It is calculated as a ratio of energy required for the probe to enter the formulation and exit from it. Another important parameter that affects the structural properties of the formulation is elasticity and is determined after the second cycle of compression and refers to the elastic recovery of the formulation after deformation [3,52].

The values referring to cohesiveness, resilience and elasticity of the tested gel samples (Gel 1, Gel 2 and Gel 3) over a 14 days period are shown in Figure 3c and Figure 3d, and Figure 3e, respectively.

The results shown in Figure 3c–e do not indicate statistically significant changes in the values of textural parameters (cohesiveness, resilience, and elasticity) of the examined gels (Gel 1, Gel 2, and Gel 3) over a 14-day period. In addition, there are no statistically significant changes in the values of these parameters among the examined gels (Gel 1, Gel 2, and Gel 3). Based on the results shown in Figure 3, it can be concluded that the incorporation of curcumin inclusion complex and curcumin into Carbopol^®^ gel did not lead to statistically significant changes in the textural characteristics of Carbopol^®^ gel, i.e., it did not negatively affect the textural properties of the gel. Measuring over 21, 28, and 35 days (Figure 4) confirmed that all textural parameters of Gel 1, Gel 2, and Gel 3 remained stable, demonstrating the long-term physical stability of the formulations. The absence of statistically significant changes in textural parameters during the 35-day period with respect to the time of preparation, can potentially imply the physical stability of the formulations (Gel 2 and Gel 3). Considering that curcumin exhibits a wide range of pharmacological activities (antioxidant, anti-inflammatory, antibacterial, etc.), the formulations developed in this way (Gel 2 and Gel 3) can be used for topical application, which conforms with the data in the reference [13,14,15].

### 2.4. Scanning Electron Microscopy (SEM)

**Figure 5:**
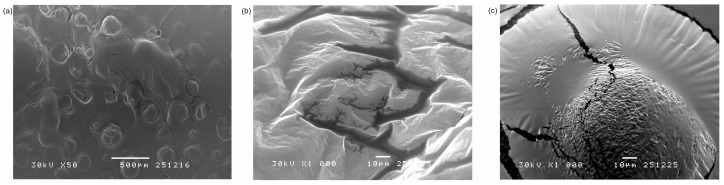
SEM micrographs of: (**a**) Gel 1, (**b**) Gel 2, and (**c**) Gel 3.

In Figure 5a, a relatively smooth and homogeneous surface of Carbopol^®^ gel can be observed, along with a large number of pores with thin, slightly wrinkled walls. In Figure 5b, a pronounced relief with folds and layered structures is observed. The gel surface is more compact and denser, with cracks integrated into the gel structure. The surface of Carbopol^®^ gel with dispersed curcumin (Figure 5c) is more heterogeneous, with visible microcracks and a pronounced granular structure in the central region. The granular structure is likely a consequence of the non-uniform dispersion of curcumin, which affects the uniformity of the gel network. Based on the observed morphologies, it can be concluded that the inclusion complex is more uniformly distributed within the gel and exhibits better interaction with the gel matrix, which is favorable for the potential controlled delivery of the active substance.

### 2.5. Swelling of Gels

**Figure 6:**
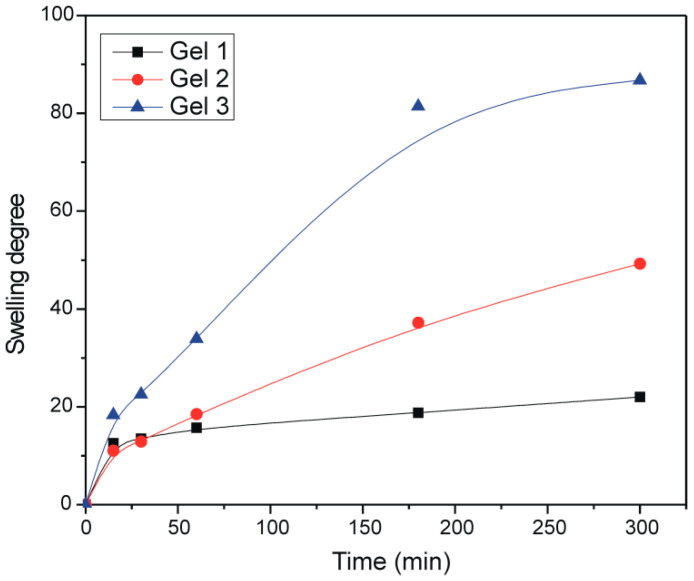
Dependence of the swelling degree of gels on the time in distilled water.

Figure 6 shows the dependence of the swelling degree on time for the formulations (Gel 1, Gel 2, and Gel 3) in distilled water at 25 °C.

From Figure 6, it can be seen that the lowest degree of swelling (α = 21.99) during the examined period is achieved by Gel 1, a higher degree of swelling (α = 49.25) is achieved by Gel 2, while Gel 3 reaches the highest degree of swelling (α = 86.79). Gel 3 shows the largest and fastest increase in the degree of swelling, while in samples Gel 1 and Gel 2 swelling increases gradually with time. After 300 min, the gels partially dissolved in water, so further monitoring of the degree of swelling as a function of time was not possible. Based on the presented results, it can be concluded that the presence of the complex partially increases hydrophilicity, while curcumin significantly increases the ability of the polymer network to absorb water. The final pH values were determined to be 5.48 for Gel 1, 5.60 for Gel 2, and 5.50 for Gel 3.

### 2.6. Rheological Evaluation

**Figure 7:**
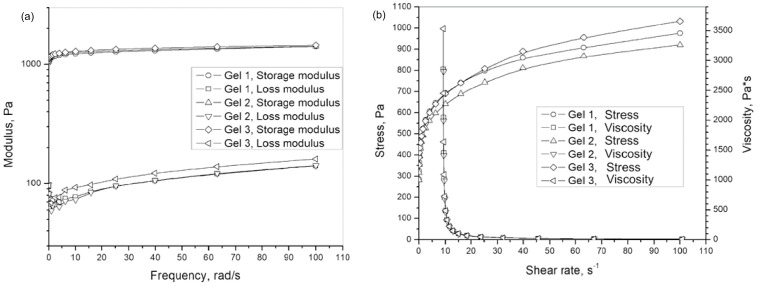
Rheological properties of gels: (**a**) Frequency dependence of storage and loss moduli, and (**b**) Dependence of stress and viscosity on shear rate.

The rheological properties of Gel 1, Gel 2, and Gel 3 are shown in Figure 7.

From the graphs shown in Figure 7, it can be observed that the presence of curcumin and inclusion complex in Carbopol^®^ gel, at the concentrations used in the experiments, does not significantly affect the rheological parameters. This indicates that the incorporation of curcumin and inclusion complex does not compromise the structural integrity of Carbopol^®^ gel.

### 2.7. The Release of Curcumin from Gels

**Figure 8:**
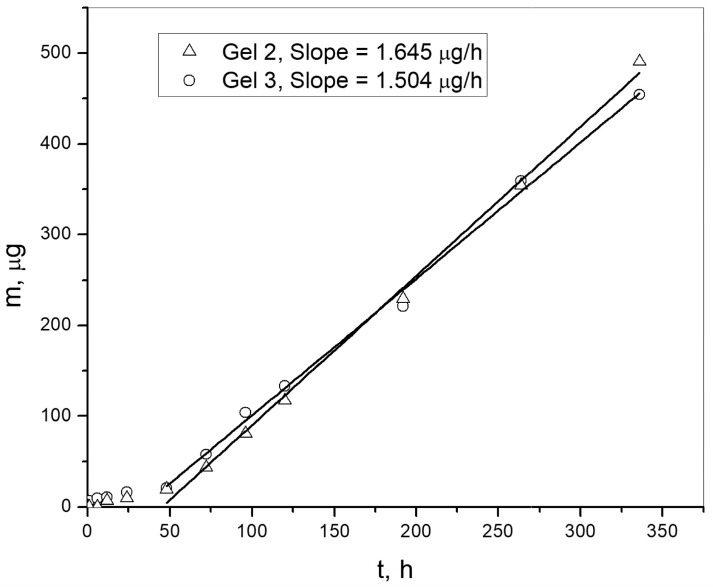
Amount of curcumin released from Gel 2 and Gel 3 samples.

The concentration of curcumin released from the gels containing the complex and curcumin into Hank’s buffer solution with 10% Tween 20 was determined using Equation (4), derived from the curcumin calibration curve. The release of curcumin from the gels was monitored under in vitro conditions at a temperature of 37 °C and pH 7.4 using the HPLC method (Figure 8). The release of curcumin from the gels was monitored for 14 days.

The release rates of curcumin from the gel with the complex (Gel 2) and from the gel with curcumin (Gel 3) were 1.645 µg/h and 1.504 µg/h, respectively, under the experimental conditions after 48 h. During the first 48 h, the release was very slow. After 48 h, a steady state was reached and the release rates became constant. When normalized to the amount of gel, the release rates were 0.414 µg/h·g_gel_ and 0.376 µg/h·g_gel_, respectively. These results indicate that the release rates are similar, although the release from the gel with the complex is slightly higher.

## 3. Conclusions

Curcumin/2-hydroxypropyl-β-cyclodextrin inclusion complex was successfully prepared using the coprecipitation method. The antioxidant activity of curcumin and curcumin/2-hydroxypropyl-β-cyclodextrin inclusion complex was tested using DPPH, ABTS, and FRAP tests. After the complexation with 2-hydroxypropyl-β-cyclodextrin, the antioxidant activity of curcumin increased. The results of evaluation of the antimicrobial activity of curcumin and curcumin in curcumin/2-hydroxypropyl-β-cyclodextrin inclusion complex indicate a stronger antimicrobial activity of curcumin in inclusion complex, which is probably a consequence of its increased solubility in water. The lowest MIC value (5 μg/cm^3^) for curcumin in inclusion complex was achieved for the *Enterococcus faecalis* strain and is approximate to the MIC value (3.91 μg/cm^3^) for the tested antibiotic Chloramphenicol and is even 156 times lower than the MIC value obtained (0.78 mg/cm^3^) for pure curcumin for the same microbial strain. The SEM analysis confirmed the presence of curcumin and inclusion complex within the Carbopol^®^ gel matrix. Rheological studies showed that the incorporation of curcumin and inclusion complex did not compromise the structural integrity of Carbopol^®^ gel. Among the tested formulations, Gel 3 exhibited the highest swelling degree, indicating enhanced water uptake. In vitro studies showed that the release of curcumin from Gel 2 and Gel 3 occurred at the rates of 0.414 µg/h·g_gel_ and 0.376 µg/h·g_gel_, respectively. Textural analysis of hydrophilic Gel 2 and Gel 3 showed that curcumin and curcumin/2-hydroxypropyl-β-cyclodextrin inclusion complex do not lead to statistically significant changes in textural parameters of Carbopol^®^ gel and that the formulations prepared in this way are physically stable for a period of 35 days. The results obtained in this study indicate the potential use of the designed formulations for topical application. Future research should focus on conducting in vivo studies, as this would validate the results obtained in vitro and further support the application of the developed formulations for topical use on the skin.

## 4. Materials and Methods

### 4.1. Material

Curcumin 97%, (Tokyo Chemical Industry Co., Tokyo, Japan); 2-hydroxypropyl-β-cyclodextrin 97%, (Tokyo Chemical Industry Co., Tokyo, Japan); ethanol 99.5%, (Merck KGaA, Darmstadt, Germany); Carbopol 940^®^ (Comcen, Belgrade, Serbia); propylene glycol (Fargon, Rotterdam, Holland); triethanolamine (Henkel, Rocky Hill, CT, USA); sodium benzoate (Merck KGaA, Darmstadt, Germany). Distilled water.

### 4.2. Preparation of Curcumin/2-Hydroxypropyl-β-Cyclodextrin Inclusion Complex

**Figure 9:**
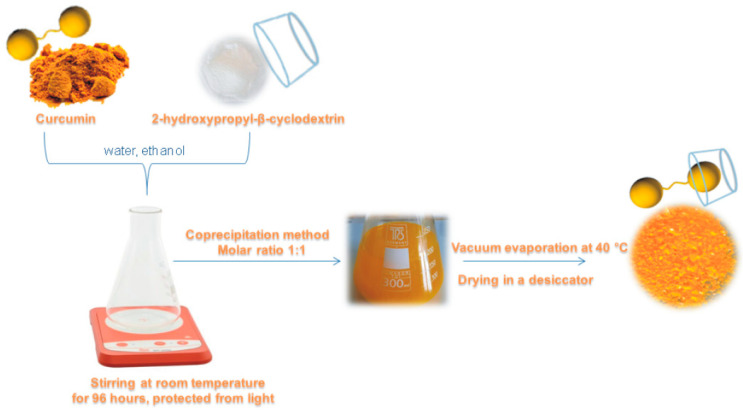
Schematic representation of inclusion complex preparation.

The inclusion complex was prepared according to the previously described procedure [39]. Curcumin (368.38 mg) was dissolved in 200 cm^3^ absolute ethanol and added to a solution of 2-hydroxypropyl-β-cyclodextrin which was prepared by dissolving 1541.54 mg of 2-hydroxypropyl-β-cyclodextrin in 100 cm^3^ distilled water. The obtained mixture was equilibrated by stirring on a magnetic stirrer at a room temperature for 96 h, protected from light. The resulting solution was concentrated on a vacuum evaporator at 40 °C to a minimum volume and then dried in a desiccator over a dehydrating agent at a room temperature until a constant mass was reached. The molar ratio of curcumin and 2-hydroxypropyl-β-cyclodextrin in the inclusion complex was 1:1. A schematic representation of the preparation of the inclusion complex is shown in Figure 9.

### 4.3. Preparation of Physical Mixture

The physical mixture is prepared by simply mixing curcumin and a complexing agent (2-hydroxypropyl-β-cyclodextrin) in a mortar with a pestle, in a 1:1 molar ratio. The physical mixture consists of a simple blend of the active substance and cyclodextrin, without forming an inclusion complex, and was prepared to confirm the incorporation of curcumin into 2-hydroxypropyl-β-cyclodextrin using various characterization methods.

### 4.4. Preparation of Hydrophilic Carbopol^®^ Gel with Curcumin and Curcumin/2-Hydroxypropyl-β-Cyclodextrin Inclusion Complex

A carbomer-based hydrogel, trademarked as Carbopol^®^, was prepared (Table 2) according to the instructions given in the 2008 Magistral formulations [53].

Carbopol 940^®^ was mixed with a small amount of purified water. The rest of the purified water was gradually added and mixed until a lump-free dispersion was formed. The dispersion was diluted with propylene glycol while stirring and a triethanolamine solution was added to neutralize it. The resulting mixture was allowed to swell until appropriate gel consistency was achieved while stirring occasionally. The gel was preserved with sodium benzoate (0.1%).

Three gel samples were prepared for the purpose of a texture analysis. The composition of the gels is given in Table 3.

Gel 1 is a sample containing only Carbopol^®^ gel prepared by following the instructions given in 2008 Master Formulas and used as a placebo in the analysis. Curcumin inclusion complex and curcumin were dispersed in two gel samples (Gel 2 and Gel 3), respectively. Gel 2 and Gel 3 samples contained the same concentration of the bioactive substance, curcumin. The gels were homogenized for 30 min using a propeller laboratory mixer RV16 basic (IKA-WERKE GmbH & Co. KG, Staufen im Breisgau, Germany). The curcumin concentration in Gel 2 and Gel 3 is identical, at 0.1% *w*/*w*. Gels 1, 2, and 3 were stored at a room temperature, protected from light, throughout the storage period. The gels prepared in this way were subjected to the textural analysis.

### 4.5. Antioxidant Activity of Curcumin and Curcumin/2-Hydroxypropyl-β-Cyclodextrin Inclusion Complex

#### 4.5.1. DPPH Test

The decolorization property of a stable 1,1-diphenyl-2-picryl hydrazyl (DPPH) radical was used to determine the capacity of the compound to scavenge free radicals in the presence of antioxidants. Some batches of solutions were prepared in ethanol with different concentrations of curcumin and curcumin/2-hydroxypropyl-β-cyclodextrin inclusion complex (0.00049–0.25 mg/cm^3^). The ethanol solutions of curcumin and curcumin/2-hydroxypropyl-β-cyclodextrin inclusion complex were protected from light. An ethanol solution of DPPH radicals (1 cm^3^, concentration 3 × 10^−4^ mol/dm^3^) was added to 2.5 cm^3^ of prepared solutions of curcumin and curcumin/2-hydroxypropyl-β-cyclodextrin inclusion complex. The absorbance was measured at 517 nm at a room temperature 20 min after incubation with a radical. The absorbance was also determined for the ethanolic solution of DPPH radicals (1 cm^3^ of DPPH radicals and 2.5 cm^3^ of ethanol) and the ethanolic solution of curcumin and curcumin/2-hydroxypropyl-β-cyclodextrin inclusion complex (2.5 cm^3^ of solution and 1 cm^3^ of ethanol). The free radical scavenging capacity was calculated using Equation (1):

(1) $$\mathrm{DPPH}\;\mathrm{radical}\;\mathrm{scavenging}\;\mathrm{capacity}\;\left(\%\right)=100-\left[\left(A_{U}-A_{B}\right)\times \frac{100}{A_{K}}\right]$$

where *A_U_*—Sample absorbance (ethanolic sample solution treated with DPPH radical) at 517 nm; *A_B_*—Blank sample absorbance (ethanolic sample solution not treated with DPPH radical) at 517 nm; *A_K_*—Control sample absorbance (diluted ethanolic solution of DPPH radical) at 517 nm.

All absorbances were measured on a UV-Vis Varian Cari100 Conc. spectrophotometer. The experiments were performed in triplicate and expressed as a mean value.

#### 4.5.2. ABTS Test

The antioxidant activity of pure curcumin and curcumin in curcumin/2-hydroxypropyl-β-cyclodextrin inclusion complex was determined using a modified ABTS test [54]. An ABTS cation radical (ABTS^•+^) is formed in the reaction of ABTS solution (7 × 10^−3^ mol/dm^3^) and potassium persulfate (2.4 mM) in a volume ratio of 1:1 *v*/*v*, within 12–18 h, in the dark, at 4 °C (basic solution). After the formation of radicals, a working ABTS solution was made by diluting the stock solution with ethanol until an absorbance value of 0.7 at 734 nm was achieved.

The batches with ethanolic solutions of different concentrations of curcumin (0.25–0.0039 mg/cm^3^) and curcumin/2-hydroxypropyl-β-cyclodextrin inclusion complex (0.5–0.0039 mg/cm^3^) were prepared. The ethanolic solutions of curcumin and curcumin/2-hydroxypropyl-β-cyclodextrin inclusion complex were protected from light. After adding 1.8 cm^3^ of ABTS working solution and 2.1 cm^3^ of ethanol to 0.1 cm^3^ of each of the prepared solutions of curcumin and curcumin/2-hydroxypropyl-β-cyclodextrin inclusion complex, the absorbance of the reaction mixture was measured at 734 nm (A_U_ value), 6 min upon incubation in the dark at a room temperature. The absorbance was measured at 734 nm for the diluted ABTS working solution (1.8 cm^3^ of working ABTS radical solution was diluted by adding 2.2 cm^3^ of ethanol, this is the Ak value) as well as for the curcumin and curcumin/2-hydroxypropyl-β-cyclodextrin inclusion complex solutions before treating them with the ABTS radical (3.9 cm^3^ of ethanol was added to 0.1 cm^3^ of the sample solution, this is A_B_ value). Ethanol was used as a blank. The percentage of ABTS radical scavenging was calculated using Equation (1).

#### 4.5.3. FRAP Test

A FRAP test was used to evaluate the antioxidant activity of curcumin and curcumin/2-hydroxypropyl-β-cyclodextrin inclusion complex, according to Benzie and Strain method [55] with certain modifications.

The standard curve: A FRAP reagent was prepared by mixing acetate buffer (300 mmol/dm^3^, pH = 3.6), TPTZ reagent (10 mmol/dm^3^ in 40 mmol/dm^3^ HCl) and FeCl_3_ × 6 H_2_O (20 mmol/dm^3^) in a 10:1:1 ratio. A volume of 3 cm^3^ of FRAP reagent and 0.1 cm^3^ of standard solution FeSO_4_ × 7H_2_O (0.2–1 mmol/dm^3^) was dispensed in each of five test tubes. The absorbance was recorded at 593 nm in relation to the blank sample (3 cm^3^ of the FRAP reagent and 0.1 cm^3^ of water). A calibration curve was constructed as absorbance dependence on the known concentration of FeSO_4_ × 7H_2_O solution [41]. The method for determining the antioxidant activity of the samples: a volume of 0.1 cm^3^ of curcumin solution and curcumin/2-hydroxypropyl-β-cyclodextrin inclusion complex (0.125 mg/cm^3^) and 3 cm^3^ of the FRAP reagent were added to each of the test tubes, and after 30 min incubation at 37 °C in a water bath, absorbance was recorded at 593 nm. The concentration of Fe^2+^ in the samples (mmol/dm^3^) was determined from the calibration curve Equation (2) and converted to mg Fe^2+^/g of the sample, i.e., FRAP value. A higher FRAP value indicates a better reducing ability of the sample.

(2) A=0.00495+0.65743×c

where *A*—absorbance at 593 nm; *c*—concentration of FeSO_4_ × 7H_2_O in mmol/dm^3^, R^2^ = 0.999.

### 4.6. Antimicrobial Activity of Curcumin and Curcumin/2-Hydroxypropyl-β-Cyclodextrin Inclusion Complex

The evaluation of the antimicrobial activity of curcumin and the curcumin in curcumin/2-hydroxypropyl-β-cyclodextrin inclusion complex was conducted against human pathogenic strains from the reference ATCC collection (American Type Culture Collection): Gram (-) bacteria—*Escherichia coli* ATCC 8739, *Pseudomonas aeruginosa* ATCC 9027, *Enterobacter aerogenes* ATCC 13048 and *Salmonella enteritidis* ATCC 13076); Gram (+) bacteria—*Staphylococcus aureus* ATCC 6538, *Enterococcus faecalis* ATCC 29212, *Streptococcus pneumoniae* ATCC 6301, *Streptococcus pyogenes* ATCC 19615, *Bacillus cereus* ATCC 11778, and yeast *Candida albicans* ATCC 24433.

#### 4.6.1. Micro-Dilution Method

The in vitro antimicrobial activity of curcumin and curcumin in curcumin/2-hydroxypropyl-β-cyclodextrin inclusion complex was examined using the micro-dilution method with some modifications [56].

Some overnight cultures of microbial strains were grown in appropriate conditions—bacteria at 37 °C on Miller-Hinton agar (MHA), and yeast at 30 °C on saburo-dextrose agar (SDA). The suspensions of 0.5 MFU turbidity (McFarland Units) were prepared from the overnight cultures of bacteria and yeast in a sterile physiological solution (0.9% NaCl), which corresponds to 108 CFU/cm^3^. The suitable culture media—Mueller-Hinton broth for bacterial strains and Sabouraud dextrose broth for yeasts were inoculated on 96-well microtiter plates with the suspensions prepared in this way. Dilution batches were made from the initial sample solutions of curcumin and curcumin/2-hydroxypropyl-β-cyclodextrin inclusion complex (in 100% DMSO) on microtiter plates at concentrations of 0.003–25.0 mg/cm^3^.

In each well of the microtiter plate, the total volume was 100 µL, whereas the final concentration of microbial cells was 106 CFU/cm^3^. The microtiter plates were cultured at 37 °C (for bacteria) and at 30 °C (for yeast) during the period of 24 h. A dilution batch of pure solvent (DMSO) was used as a negative control, whereas the batches of antibiotic dilution (0.02–10 µg/cm^3^)—tetracycline and streptomycin (for bacteria) and nystatin (for yeasts) were used as positive controls. The inhibitory activity of the samples was determined by using the method of visible growth, i.e., by adding 20 µL of 0.5% aqueous triphenyl tetrazolium chloride (TTC) to each well of the microtiter plate.

The minimum inhibitory concentration (MIC) was defined as the lowest concentration of samples that inhibited visible growth of microorganisms (red color appearing after adding TTC). In order to determine minimum bactericidal/fungicidal concentrations (MBC/MFC), the contents from each well were transferred to Petri plates with appropriate media and incubated under the specific conditions described for the tested microbial strains. MBC/MFC was defined as the lowest concentration of the tested samples whereupon the inoculated microorganisms were 99.9% killed. All experiments were performed in triplicate.

#### 4.6.2. Statistical Data Processing

For these analyses, a statistical analysis based on the analysis of variance (ANOVA) was applied to determine the significance (*p* ≤ 0.05) of the data obtained from all experiments. All results were found to be at 95% reliability level for the reproducibility of the experiment. For these assays, a statistical analysis was applied using the IBM SPSS Statistics 22 software package to determine the significance (*p* ≤ 0.05) of the data obtained in all assays.

### 4.7. Texture Analysis of Carbopol^®^ Gel with Curcumin and Curcumin.2-Hydroxypropyl-β-Cyclodextrin Inclusion Complex

The textural properties of the samples were evaluated using a CT3 Texture Analyzer texturometer (Brookfield, AMETEK Inc., Middleboro, MA, USA) and a TA-STF cone probe using the Texture Profile Analysis (TPA) test. The analysis parameters are

- Load cell: 10 kg
- Probe speed: 1.5 mm/s
- Deformation: 5 mm
- Initial load: 50 mN.

The samples were placed in sample containers (75% of the total container volume was filled, no air present). During the TPA test, two cycles of compression were performed on the samples and five texture profiles were measured: hardness, cohesiveness, adhesiveness, resilience, and elasticity. For each sample, three measurements were taken at a room temperature.

### 4.8. Scanning Electron Microscopy (SEM)

The morphology of Gel 1, Gel 2, and Gel 3 was examined by scanning electron microscopy. Before the analysis, a dried sample was coated with gold/palladium alloy (15/85) by using a sprayer JEOL Fine Coat JFC 1100E Ion Sputter (JEOL Ltd., Tokyo, Japan) and recorded on apparatus JEOL Scanning Electron Microscope JSM-5300 (JEOL Ltd., Tokyo, Japan).

### 4.9. Swelling of Gels

The swelling of the dried gels (Gel 1, Gel 2, and Gel 3) was monitored gravimetrically. A defined amount of each gel was immersed in an aqueous solution at pH 7.4 and 25 °C, and the sample mass was measured at specific time intervals until equilibrium was reached. The degree of swelling, α, was calculated according to Equation (3):

(3) $$\alpha =\frac{m-m_{0}}{m_{0}}$$

where *m*_0_—is mass of dry gel, *m*—mass of swollen gel in a point of time *t*.

The pH values of Gel 1, Gel 2, and Gel 3 were measured using a pH meter (HI9318-HI9219, Hanna Instruments, Padua, Italy).

### 4.10. Rheological Evaluation

Shear rate viscosity dependences of samples were recorded on a Discovery Hybrid Rheometer HR2 (TA Instruments, New Castle, DE, USA). Parallel plate geometry was used with a plate diameter of 25 mm (gap 1 mm) and with a Peltier system for temperature control. Rheological data were collected in the shear rate range of 0.1–100 s^−1^ (with 5 points per decade) at 25 °C. Averaging and equilibration times at each shear rate point were set at 10 s.

The viscoelastic properties of gels were studied by a dynamic mechanical analysis in the shear mode. The experiments were performed on a Discovery Hybrid Rheometer HR2 (TA Instruments, New Castle, DE, USA) in parallel plate geometry with a plate diameter of 25 mm and equipped with a Peltier device for temperature control. Oscillatory sweep tests with varying frequency from 0.1 to 100 rad s^−1^ at 25 °C were performed. The strain amplitude was kept constant for all samples at 3%, which was ascertained to be in the linear viscoelastic range while the gap was set at around 1 mm. The storage (G’) and loss modulus (G”) frequency curves were analyzed.

### 4.11. Release of Curcumin from Gels

In vitro study of the release of curcumin from gels with dispersed curcumin and inclusion complex was carried out in a medium that simulates physiological conditions. Each sample (4 g) in Dialysis sacks (35 mm, 12 kDa MWCO, Sigma-Aldrich, St. Louis, MO, USA) was covered with 30 cm^3^ of solution (27 cm^3^ Hank’s BSS buffer with pH value 7.4 and 3 cm^3^ of Tween 20 solution). The samples were thermostated in a water bath at 37 °C while stirring on a magnetic stirrer (Magnetic stirrer HI 190M, Hanna Instruments, Padua, Italy) over a period of 14 days. The amount of curcumin released was monitored by sampling 200 μL of solution over time, which were then filled up with methanol up to 1 cm^3^, filtered on a cellulose membrane filter with a pore diameter 0.45 μm and analyzed by HPLC method.

### 4.12. Determination of the Concentration of Curcumin by Using High Pressure Liquid Chromatography (HPLC)

Monitoring of curcumin release from the gels was performed by using liquid chromatography HPLC method (The HPLC Agilent 1100 Series with diode-array detector, DAD 1200 Series, Agilent Technologies, Santa Clara, CA, USA) under these conditions: column Zorbax Eclipse XDB-CN 250 × 4.6 mm, 5 μm (Agilent Technologies, Inc., Santa Clara, CA, USA); eluent was methanol: mobile phase flow was 1 cm^3^/min; the volume of injected samples 20 μL; column temperature 40 °C; detection wavelength 425 nm. For the straight line part of the curcumin calibration curve in range 0.50–25 μg/cm^3^, Equation (4) applies with a linear correlation coefficient R^2^ = 0.999.

(4) $$c=\frac{A-21.07}{95.83}$$

where A is the peak curcumin area (mAU·s), and c is the concentration of curcumin (μg/cm^3^).

## Figures and Tables

**Table 1:** Antimicrobial activity of curcumin and the curcumin in curcumin/2-hydroxypropyl-β-cyclodextrin inclusion complex.

Tested Samples		Curcumin	Curcumin in the Complex	Antibiotics
Microbial Strains	Source	MIC/MBC (mg/cm^3^)	MIC/MBC (mg/cm^3^)	MIC/MBC (μg/cm^3^)
Gram (+) bacteria	ATCC			Chloramphenicol
*Staphylococcus aureus*	6538	0.39/25.0	0.037/4.82	7.81/15.61
*Enterococcus faecalis*	29212	0.78/25.0	0.005/4.82	3.91/7.81
*Streptococcus pneumoniae*	6301	1.56/>25.0	0.134/>4.82	0.06/0.12
*Streptococcus pyogenes*	19615	0.39/25.0	0.037/4.82	0.25/0.49
*Bacillus cereus*	11778	0.19/>25.0	0.037/>4.82	7.81/15.61
Gram (-) bacteria	ATCC			Streptomycin
*Pseudomonas aeruginosa*	9027	12.5/>25.0	0.037/>4.82	0.60/0.60
*Salmonella enteritidis*	13076	0.78/>25.0	0.075/>4.82	0.30/0.30
*Escherichia coli*	8739	12.5/>25.0	0.037/>4.82	0.16/0.16
*Enterobacter aerogenes*	13048	12.5/>25.0	0.134/>4.82	0.60/0.60
Fungus	ATCC	MIC/MFC (mg/cm^3^)	MIC/MFC (mg/cm^3^)	Nystatin (μg/cm^3^)
*Candida albicans*	24433	25.0/>25.0	0.037/>4.82	3.91/7.81

**Table 2:** Gel composition.

Components	Function	Gel Composition (%, *w*/*w*)
Carbopol 940^®^	Gelling agent	1.00
Propylene glycol	Humectant	10.00
Triethanolamine, 10%	Neutralizer	14.00
Purified water	Water phase	100

**Table 3:** Types and amount of the components in the composition of gels.

	Gel 1 (g)	Gel 2 (g)	Gel 3 (g)
Curcumin			0.2
Curcumin inclusion complex		1.3	
Carbopol^®^ gel	to 200	to 200	to 200

## Data Availability

The data presented in this study are openly available in article.
